# Household Hints and Recipes

**Published:** 1882-10

**Authors:** 


### Household Hints & Recipes.

STAIN FOR MAHOGANY CHERRY.—The most simple and best stain for mahoganizing cherry is ground burnt sienna, mixed in benzine or turpentine. Apply with a brush or sponge, let it stand for a short time, and clean off with a cloth. It will be better to let it remain in this condition until the following day before commencing to finish.

RED WRITING FLUID.—

Eosine, 10 grains.
Water, 1 ounce.

A good red ink for rubber stamps is obtained by a slight modification as follows :

Eosine 15 grains.
Glycerine 4 drachms.
Water 4 drachms.

AQUEOUS SHELLAC VARNISH.—Dr. Geissler, in the *Pharm. Centralhalle*, recommends as a varnish for maps, etc. :

Saturated solution of borax, 2 parts.
Powdered shellac, 1 part.

Shake together but apply no heat. The shellac will dissolve in two or three days. He says this varnish also forms an excellent *starch gloss*.

FIRE AND WATER PROOF PAPER —Mix one-third of ground asbestos fibre with two-thirds of paper paste in a solution of common salt and alum. Pass the mixture into a bath of dissolved gum lac and send it through the finishing rolls, when it may be cut into sheets. The salt and alum increase the strength of the paper and its resistance to the action of fire. The lac renders it impermeable to moisture without interfering with its fitness for the reception of ink.

TO IMITATE BLACK WALNUT.—A new process by which ordinary wood has imparted to it the appearance of walnut, suitable for office, steamboat, and other cabinet work, has been recently described. Birch, beech, alder, or similar woods, are first thoroughly dried and warmed, then coated once or twice with a liquid composed of one part by weight of extract of walnut peel, dissolved in six parts of soft water, by heating it to boiling and stirring. The wood thus treated is, when half dry, brushed with a solution of one part by weight of bichromate of potash in five parts of boiling water, and, after drying thoroughly, is rubbed and polished.

TO PRESERVE IRON FROM RUST.—M. Ventura Lerra, after many years of experiment and observation, having noticed that knives used in cutting plants, belonging to the family of Euphorbiaceæ did not rust, is led to recommend for this purpose an alcoholic solution of gum (resin of) euphorbium. This when applied to steel, iron, or copper forms a thin, uniform, and very adherent layer, which effectually protects the metal. Experiments with copper immersed in sea-water—a ship's sheathing—were followed by gratifying results.

A STRONG CEMENT FOR MICA, GLASS OR METALS, ETC.—Take equal weights of Nelson's crystal gelatine, and of cut Penang isinglass, and allow them to macerate in cold distilled water for at least 24 hours. Drain the expanded gelatinous shreds thoroughly, first in a colander, and then in an absorbent cloth. Dissolve the whole in the smallest possible quantity of spirits of wine—methylated spirit free from gum or color will do—of not less than 54° over-proof, by the aid of gentle heat.

To every 10 fluid ounces of this solution, add—previously dissolved in spirit containing 5 per cent. of acetic ether—1 drachm of mastic, 3 of sandarac, and 2 of ammoniacum, taking care to mix the whole very perfectly before pouring into bottles and putting aside to cool.

In using this cement it is merely necessary to render it fluid by a gentle heat, and to warm the surface intended to be united before applying the cement to *both*, pressing them together and wiping off any superfluity. In 24 hours time the joint will sustain any moderate amount of force without giving way, and after two or three days a severe strain, or even hot liquids may be applied without injury.

In illustration of this, a desert plate was broken into three pieces, of which two were strongly riveted together, and the third cemented as above. A force of 167 lb. secured a fracture, chiefly where the rivets were, tearing them away, but the cemented portion remained as sound as when first joined.

A GOOD CEMENT FOR GLASS.—A solution of bichromate of potassium and glue yields a superior cement for broken glassware. The moderately strong glue or gelatin solution is mixed, in a dark place or in a photographic dark-room, with a small amount of concentrated solution of bichromate of potassium. The edges of the fracture, which have been thoroughly cleaned, are then coated with a thin layer of the mixture, strongly pressed together, and kept close by tying with twine, or in some other manner. The glass is then exposed to sunlight for some hours. This causes the cement to become insoluble even in hot weather.

---

TO REMOVE FRECKLES.—

Oil of almonds, expressed, 4 oz.
Lard, 3 oz.
Spermaceti, 1 oz.
Expressed juice of houseleek, 3 fl. oz.

Melt the spermaceti and lard together; add the oil and then the juice, and stir the mixture until it solidifies on cooling. A few drops of some perfume, as cologne, may be added.—*Oil and Drug News.*

---

CEMENT FOR GLASS AND METAL. — Every one who uses brass letters on glass windows, and knows how often they drop off from unequalled expansion or from the too energetic efforts of window-cleaners, will be glad to have the following receipt:

Litharge, 2 parts.
White lead, 1 part.
Boiled linseed oil, 3 parts.
Gum copal, 1 part.

Mix just before using. This forms a quick drying and secure cement.— *Chemists' Journal.*

---

ALGERIAN CORN CURE.—Dr. Barbier speaks highly of the subjoined:

Acetic acid, 1 ounce.
Iodine, 37 grains.
Alcohol, 1 ounce.

Dissolve the iodine in the alcohol and add the acetic acid. A few drops of the liquor are to be rubbed on the corn morning and evening, so as to gradually dissolve it.

---

PASTE FOR MOUNTING PHOTOGRAPHS.—Mix thoroughly 630 grains of the finest Bermuda arrowroot with 375 grains of cold water in a capsule, with a spoon or brush, then add 10½ ounces more water and 60 grains of gelatine in fine shreds. Boil, with stirring, for five minutes, or until the liquid becomes clear, and when cold, stir in well 575 grains of alcohol and five to six drops of carbolic acid. Keep it in well closed vessels, and before using work up a portion carefully with a brush in a dish. It will keep for a considerable time.

---

SALICYLIC ACID TO PRESERVE CIDER should be added in the free state—that is, uncombined with any of the substances usually employed as solvents for its administration in medicine. The quantity needed, from one-quarter to one-half an ounce to a barrel of cider, can easily be taken up by this amount of liquid without other addition. All that is necessary is to agitate thoroughly to insure complete solution of the acid. In case sulphate of lime is also used, the quantity of acid should be reduced in proportion.

---

TO DESTROY ROACHES.—Probably the best way is to blow into the crevices where they mostly congregate a mixture of equal weights of powdered borax and Persian insect powder. The operation is to be repeated frequently, but, with a little perseverance, the insects can be after awhile completely destroyed. Another method, employed by some with reported success, is to feed the roaches regularly every night with a mixture of flour, water, and phosphorus paste. We would prefer the first way, because no poisons are employed, but phosphorus paste has a taste and appearance so easily recognizable that scarcely any danger need be apprehended from its use.

---

PREPARATION OF ARTIFICIAL SLATE.--We have lately noticed a German patent, granted to A. Rosenbach, in Berlin, which seems to contain a solution of the problem. The object of this inventor is to produce upon paste-board, wood, paper, tin, or leather a watertight hard coating *for writing upon it.* He directs to dissolve 3 kilos of sandarac and 3 kilos of shellac in 40 litres of 90 per cent. alcohol; then to add 6 kilos of diamond-emery, 1.5 kilo of lampblack and 0.3 kilo of ultramarine. The mass having been thoroughly stirred, it is applied to the object and ignited, whereby the resinous ingredients melt together.

---

Dry buckwheat flour, if repeatedly applied, will entirely remove the worst grease spots on carpets or any other woolen cloth, and will answer as well as French chalk for grease spots on silk.

